# Spatial and Temporal Dynamics and Molecular Evolution of *Tula orthohantavirus* in German Vole Populations

**DOI:** 10.3390/v13061132

**Published:** 2021-06-11

**Authors:** Sabrina Schmidt, Daniela Reil, Kathrin Jeske, Stephan Drewes, Ulrike M. Rosenfeld, Stefan Fischer, Nastasja G. Spierling, Anton Labutin, Gerald Heckel, Jens Jacob, Rainer G. Ulrich, Christian Imholt

**Affiliations:** 1 Institute of Novel and Emerging Infectious Diseases, Friedrich-Loeffler-Institut (FLI), Federal Research Institute for Animal Health, 17493 Greifswald-Insel Riems, Germany; sabrina05schmidt@gmail.com (S.S.); kathrin.jeske@fli.de (K.J.); stephan.drewes@fli.de (S.D.); ulrike.rosenfeld@gmx.de (U.M.R.); stefan.fischer25@web.de (S.F.); NasiK@gmx.de (N.G.S.); RainerGuenter.Ulrich@fli.de (R.G.U.); 2 Animal Ecology, Institute of Biochemistry and Biology, University of Potsdam, 14469 Potsdam, Germany; reil@uni-potsdam.de; 3 Institute of Ecology and Evolution, University of Bern, 3012 Bern, Switzerland; anton.labutin@iee.unibe.ch (A.L.); gerald.heckel@iee.unibe.ch (G.H.); 4 Institute for Plant Protection in Horticulture and Forests, Julius Kühn-Institute (JKI), 48161 Münster, Germany; jens.jacob@julius-kuehn.de

**Keywords:** rodents, hantavirus, monitoring, population dynamics, common vole, field vole, water vole, phylogeny, molecular evolution

## Abstract

Tula orthohantavirus (TULV) is a rodent-borne hantavirus with broad geographical distribution in Europe. Its major reservoir is the common vole (*Microtus arvalis*), but TULV has also been detected in closely related vole species. Given the large distributional range and high amplitude population dynamics of common voles, this host–pathogen complex presents an ideal system to study the complex mechanisms of pathogen transmission in a wild rodent reservoir. We investigated the dynamics of TULV prevalence and the subsequent potential effects on the molecular evolution of TULV in common voles of the Central evolutionary lineage. Rodents were trapped for three years in four regions of Germany and samples were analyzed for the presence of TULV-reactive antibodies and TULV RNA with subsequent sequence determination. The results show that individual (sex) and population-level factors (abundance) of hosts were significant predictors of local TULV dynamics. At the large geographic scale, different phylogenetic TULV clades and an overall isolation-by-distance pattern in virus sequences were detected, while at the small scale (<4 km) this depended on the study area. In combination with an overall delayed density dependence, our results highlight that frequent, localized bottleneck events for the common vole and TULV do occur and can be offset by local recolonization dynamics.

## 1. Introduction

Tula orthohantavirus (TULV) is a European hantavirus that was initially discovered in the common vole (*Microtus arvalis*) and the sibling vole (*M. levis*, previously *M. rossiaemeridionalis*) [1,2]. In addition, TULV was detected in other vole species, such as field vole (*M. agrestis*), European pine vole (*M. subterraneus*), narrow-headed vole (*M. gregalis*), Major’s pine vole (*Microtus majori*) and water vole (*Arvicola* spp.) [3,4,5,6,7,8,9]. These multiple molecular surveys confirmed the role of the common vole as the major reservoir, with a usually low to medium prevalence [9]. Infections in voles other than the common vole seem to reflect spillover infections [9], although in rare cases the field vole may represent an alternative reservoir [6]. TULV-related viruses have been identified in various other *Microtus* species in Eurasia [10,11,12,13,14].

TULV contains a trisegmented RNA genome of negative polarity with the small (S) segment encoding the nucleocapsid (N) protein, but also a putative non-structural (NSs) protein with interferon antagonist properties [15]. The medium (M) segment encodes a glycoprotein precursor that is co-translationally cleaved into two glycoproteins, whereas the large (L) segment encodes an RNA-dependent RNA polymerase with several enzymatic functions [16]. Based on nucleotide sequences, genetically divergent TULV clades have been identified that partially reflect the association to evolutionary lineages in the common vole in Central Europe [9,17,18].

TULV is commonly described as non-pathogenic to humans, with very few cases of human infections or of seroconversion being reported [16,19,20,21,22]. TULV-reactive antibodies have been detected in forestry workers in Brandenburg, eastern Germany [20]. A hospitalized patient with symptoms of hemorrhagic fever with renal syndrome from the same federal state was shown to have neutralizing antibodies specific for TULV [23]. Further, in an immune-compromised patient from the Czech Republic TULV RNA was detected [21]. Recently, a human TULV infection with acute kidney injury was detected in Germany [24]. 

The common vole is widely distributed in Central Europe and as the most abundant mammal species it predominately inhabits natural and agricultural grassland habitats [25]. Apart from seasonal changes in population size, this species is known to undergo multiannual fluctuation (outbreaks) [26] that are correlated to weather conditions [27,28] and habitat factors [29]. Outbreak maxima exceed 2000 individuals/ha [30] and are observed about every 3–5 years [31]. While large-scale, synchronous outbreaks have been reported for Europe [32], cyclicity itself does not appear to be synchronous over the whole distribution range. For many rodent-borne pathogens, reservoir density-dependent transmission is a key feature of pathogen circulation as increasing host density theoretically promotes human incidence [33]. In addition, there is evidence of a strong interaction between host population dynamics, hantavirus circulation and subsequent molecular evolution. For Puumala orthohantavirus (PUUV) transmitted by bank voles (*Myodes glareolus*, formerly *Clethrionomys glareolus*) this includes seasonal and annual density dependence of pathogen circulation within the rodent host [34,35,36,37]. To date, there is little known about similar interactions in common vole populations and TULV. Here, we present the results of a longitudinal study in four regions of Germany assessing TULV prevalence and nucleotide sequence evolution in fluctuating common vole populations. We hypothesize that within common vole populations TULV prevalence is positively correlated with abundance. Additionally, we hypothesize that TULV sequence similarity reflects the association with evolutionary lineages of the common voles and is negatively correlated to increasing spatial distance between the sites, indicating that factors limiting dispersal between populations are key drivers of local molecular virus evolution.

## 2. Materials and Methods

### 2.1. Rodent Trapping and Sample Collection 

**Figure 1:**
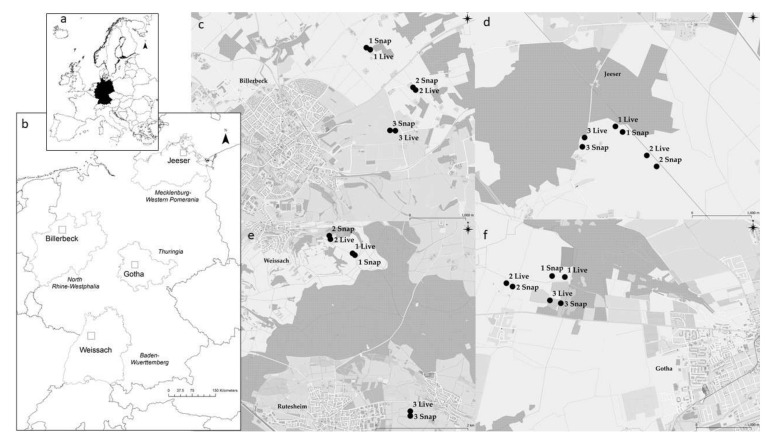
Map of the four study areas in Germany ((**a**), overview) and the corresponding federal states ((**b**), grey). In each area (Billerbeck (**c**), Jeeser (**d**), Weissach (**e**), Gotha (**f**)), trapping was conducted on three replicate sites (1, 2, 3) where live (Live) and snap (Snap) trapping was performed. Dark-grey areas present forests and light-grey areas are agricultural/grassland areas where the trapping was performed.

Voles were collected during 2010 to 2013 in spring, summer and autumn in four study areas in Germany: Jeeser (54°9.75′ N, 13°15.55′ E, Mecklenburg-Western Pomerania), Gotha (50°57.38′ N, 10°39.13′ E, Thuringia), Billerbeck (51°59.63′ N, 7°18.99′ E, North Rhine-Westphalia) and Weissach (48°49.88′ N, 8°57.71′ E, Baden-Wuerttemberg) (Figure 1). Trapping was conducted on permanent grasslands used mainly for silage production. Within each study area, three replicate sites were established in close proximity (<4 km), and within each site both live and snap trapping were performed (around 200 m apart). During trapping specific biosafety measures were followed, including wearing protective clothing, gloves and a FFP3 mask.

The snap trapping followed a standard protocol (see APHAEA standard protocol; http://www.aphaea.org/cards/species/voles, accessed on 12 October 2018). At each site, a grid of 7 × 7 traps with 10 m inter-trap distance was used and traps were baited with raisins. Rodent dissection and the collection of lung and other tissue samples followed previously established standard protocols [38]. The chest cavity was rinsed with 1 mL phosphate-buffered saline (PBS); the resulting chest cavity lavage (CCL) samples were used for detection of TULV-reactive antibodies. The dissection was performed within a BSL-3 containment dissection hall following standard hygiene and personal protection instructions.

Live trapping was conducted using the same general set-up with Ugglan live traps following procedures described previously [35]. In brief, traps were pre-baited for three days and checked twice a day for 2–3 consecutive days. Trapped animals were sexed and weighed using a 50 g spring scale (PESOLA AG^®^, Schindellegi, Switzerland). After species determination, voles were marked with a passive integrated transponder (PIT) tag (LUX-IDent s.r.o.^^®^^, Lanškroun, Czech Republic) for individual identification. Small ear pinna tissue samples were collected and stored in 80% ethanol. Blood samples (20–40 μL) were collected using the *Vena facialis* or the retro-orbital sinus and stored at −20 °C until analysis for TULV-reactive antibodies. After processing, animals were released at the point of capture. Animals found dead in live trapping were subjected to dissection as described above.

Relative abundance indices as individuals per 100 trapping nights (individuals/100TN) were calculated for both trapping methodologies (see Table S1). A comparison of abundance indices from live and snap trapping showed a significant positive linear correlation (F = 183.8, *p* ≤0.001, r^2^ = 0.82). Thus, we combined live and snap trapping data per site. This increased the number of sites where TULV prevalence could be calculated, even during years/seasons with generally low host abundance.

### 2.2. Nucleic Acid Isolation

The RNA extraction of lung tissue was performed using a modified QIAzol extraction protocol [7]. DNA was obtained from tissue samples using conventional chloroform DNA extraction or tissue lysis overnight using ear pinna or tail tissue samples [9,39].

### 2.3. Molecular Species and Sex Determination

Morphological species determination using a species determination key [40] was confirmed for all animals who tested positive by a mitochondrial cytochrome *b* (cyt *b*) gene-specific PCR [41]. In addition, for selected common voles, the mitochondrial DNA lineage in the species was determined as described before [9,42]. In case of missing morphological sex determination, sex was identified via PCR according to standard protocols [43,44].

### 2.4. TULV Detection

Detection of TULV-reactive antibodies in blood samples from live trapping as well as CCL samples from snap trapping with IgG ELISA followed previously published protocols using the yeast-expressed recombinant N protein of the TULV strain Moravia [6,20]. Hantavirus RT-PCR investigations of lung tissue samples from snap trapping followed previously described protocols for the PUUV/TULV S segment [45]. In addition, partial M and L segment sequences were determined after RT-PCR using the primers C1m (5′-CCAGCTGATTGCCCAGGGGTAG) and C2m (5′-CCTACTCCTGAGCCCCATGC; modified from [6]) and Han LF1 (5′-ATGTAYGTBAGTGCWGATGC) and Han LR1 (5′-AACCADTCWGTYCCRTCATC; [46]).

### 2.5. Sequence Determination and Phylogenetic Analyses

Sequence determination was performed by direct sequencing of RT-PCR products following a dideoxy-chain termination method using BigDye Terminator v1.1 kit (Applied Biosystems^^®^^, Darmstadt, Germany) and Genetic Analyser 3130 and 3130xl sequencing machines (Applied Biosystems^^®^^).

All generated sequences were subjected to a BLAST search-mediated comparison with sequences available in GenBank [47]. All TULV sequences were included in subsequent phylogenetic analysis. For common vole lineage analysis, three to four common voles from every trapping location were chosen for cyt *b* gene determination. Identical sequences were excluded from further analysis. Additional to the novel sequences obtained in this study, TULV sequences representative for the clades Central North (CEN.N), Eastern North (EST.N), Central South (CEN.S) and Eastern South (EST.S) were obtained from GenBank [47] and were labeled with accession numbers in Figure S1. The final datasets used for analysis contained 25 S segment sequences of 575 nucleotides (nt) length from the trapping sites Jeeser (*n* = 7) and Gotha (*n* = 8) and sequences of 572 nt length from the trapping sites Billerbeck (*n* = 3) and Weissach (*n* = 7), 21 M segment sequences of 618 nt length and 26 L segment sequences of 411 nt length for TULV and 14 sequences of 825 nt length from the cyt *b* gene of the common voles. Reference sequences for cyt *b* analysis were chosen according to [9]. 

Alignments were constructed in Bioedit (V7.2.3.) [48] using the Clustal W Multiple Alignment algorithm implemented in the program. Identical sequences were discarded from the alignment (see Table S6). The tree reconstructions were done via CIPRES [49] using partial S segment sequences of TULV (alignment length 549 nt, positions 406–951, counting according to TULV S segment, accession number NC_005227), partial M segment sequences of TULV (alignment length 348 nt, positions 2537–2884, counting according to TULV M segment, accession number NC_005228) and partial L segment sequences of TULV (alignment length 327 nt, positions 2983–3309, counting according to TULV L segment, accession number NC_005226).

Consensus phylogenetic trees of partial S, M and L segment sequences were generated by Bayesian analyses with 1 × 10^7^ generations and a burn-in phase of 25%, and maximum-likelihood analyses were performed with 1000 bootstraps and 50% cut-off using the general time-reversible (GTR) substitution model with invariant sites and a gamma-distributed shape parameter for both algorithms. 

### 2.6. Isolation-by-Distance Analysis

We tested for isolation-by-distance patterns within and between the study regions based on S segment sequences and capture location information. Isolation-by-distance represents a positive association between genetic differences and spatial distance that establishes over time if dispersal occurs only at a local scale and the accumulation of mutations in viral strains is largely restricted to the local population [50]. Genetic distances between all pairs of sequences from the study sites were estimated in MEGA version X [51]. Spatial distances between the capture locations were determined with the *geosphere* package [52] in the R software [53]. Mantel tests were performed using the *ade4* package [54] and were used to assess statistical significance of the association between genetic and spatial distances. 

### 2.7. Statistical Analysis

Differences in vole abundance as well as TULV seroprevalence between seasons, years and areas were analyzed by univariate analyses of variance (ANOVA) with subsequent post hoc tests (Tukey’s HSD). Vole abundance or TULV seroprevalence were the dependent variables, and season, year and study area were fixed factors. Analyses were performed using α < 5% as a level of significance.

A generalized linear mixed model (GLMM) with binomial distribution and a logit link function was used to statistically analyze the correlation of the common vole abundance index with TULV seroprevalence (level of significance α < 5%). The proportional response variable (two-vector variable) *TULV seroprevalence* was generated from the number of TULV-seropositive common voles and the number of TULV-seronegative common voles. The effects of the abundance index (direct effect) and the abundance index of the previous season (delayed effect), both in interaction with study area (factorial variable), were analyzed in two separate models. In each case, the trapping site nested in the study area was included as a random factor to account for the spatial and temporal design of the study. Analysis of deviance was performed to establish the overall significance of the categorical factors with more than two levels (study area). Overdispersion was checked using package *blmeco* [55] and function *dispersion glmer*. The number of paired observations of common vole abundance and TULV prevalence was *n* = 43. All analyses were done using R [53].

## 3. Results

### 3.1. Rodent Trapping

From 2010–2013 a total of 1487 common voles were caught (Table S1), and samples for TULV detection could be derived from 1304 individuals. Overall, 1062 common vole samples were derived from live trapping, and parallel snap trapping resulted in the collection of an additional 242 individuals (Table 1). In addition to common voles, a total of 180 field voles were trapped (Table S2). 

**Figure 2:**
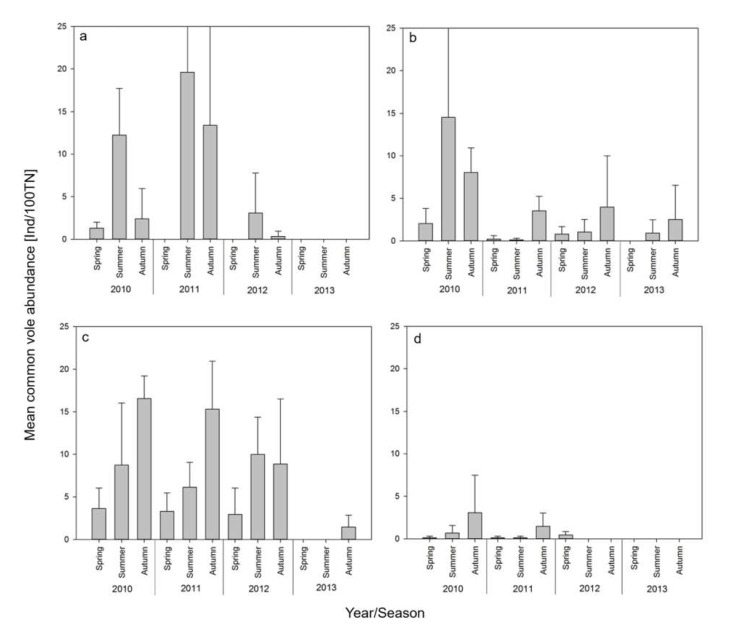
Population dynamics of common voles from 2010 to 2013 in four areas in Germany ((**a**): Weissach; (**b**): Jeeser; (**c**): Gotha; (**d**): Billerbeck)). Estimated mean abundance indices ± standard deviation as individuals per 100 trapping nights from three replicate grassland sites per area are based on live and snap trapping (see Table S1).

Site-specific common vole abundance estimates ranged from 0 to 46 individuals/100TN. Large variation between the three replicate sites of each area was observed (Table 1, Figure 2). The highest average common vole abundance was 20 individuals/100TN observed in Weissach during summer 2011 (Figure 2). 

There were significant differences in abundances among study areas (ANOVA: F = 5.83, *p* < 0.001). More precisely, abundances of common voles were significantly lower in Billerbeck than in Gotha and in Weissach (Tukey’s HSD: *p* < 0.001 and *p* = 0.027, respectively). A further statistical difference was found among seasons (F = 6.97, *p* = 0.001). Abundances were significantly lower in spring than in summer and autumn (Tukey’s HSD: *p* = 0.005, respectively). There was also a difference among years (F = 2.91, *p* = 0.038) with abundances in 2010 tending to be higher than in 2013 (Tukey’s HSD: *p* = 0.064).

Cytochrome *b* sequence analysis of 3–4 common voles from each trapping site confirmed the exclusive presence of the Central evolutionary lineage (Figure S1; for accession numbers see Table S3).

### 3.2. TULV Seroprevalence 

Overall, 9% (119) of 1304 common voles had TULV-reactive antibodies. Most seropositive individuals were found in Jeeser (14%), Gotha (7.7%) and in Weissach (7.3%) while in Billerbeck, only two individuals were seropositive (Table 1).

The mean seroprevalence per site ranged between 0% and 28.0% with the highest prevalence found in Jeeser in autumn 2012. Statistically, mean seroprevalence over the study period did not vary among study areas (ANOVA: F = 1.80, *p* > 0.05), seasons (F = 0.22, *p* > 0.05) or years (F = 1.02, *p* > 0.05). In a few cases, seroprevalence decreased from spring to summer and from summer to autumn. This could be observed in 2010 in Weissach and in 2012 in Gotha. In Jeeser, TULV-reactive antibodies were predominantly found in autumn. TULV-reactive antibodies were also detected in field voles, collected in Weissach, Jeeser and Gotha (Table S2).

Female common voles were more frequently captured than males (male:female = 1:1.2). There was an overall difference between sexes, with females being significantly less frequently seropositive compared to males (χ^2^ =4.73, *p* = 0.03).

### 3.3. Relationship of TULV Seroprevalence with Common Vole Abundance 

**Figure 3:**
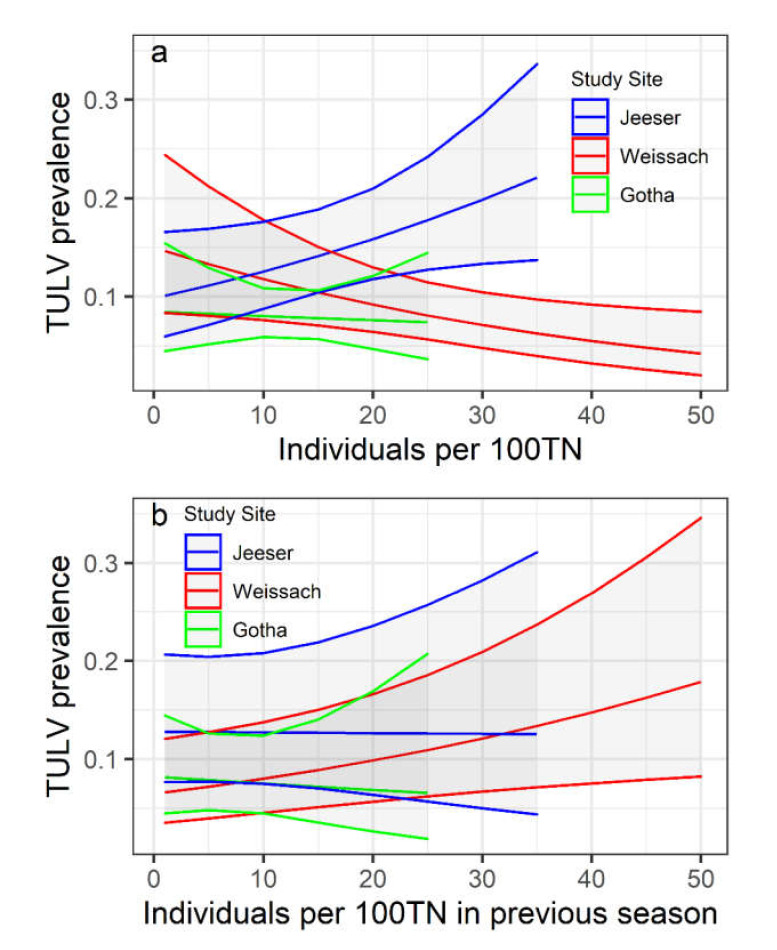
(**a**) Direct and (**b**) delayed effects of common vole abundance (as index with individuals per 100 trap nights) per study area on TULV seroprevalence in the host population.

Due to low sample sizes in Billerbeck, this area was excluded from further analysis regarding TULV seroprevalence in common voles. Linear mixed modelling revealed varying impact of direct or delayed abundance on TULV seroprevalence (Table 2). There was an overall effect of abundance on TULV prevalence, which differed for direct and delayed dependence on abundance (Table 2). The abundance in the current season was negatively associated with TULV prevalence. Analysis of deviance on multi-level categorical factors (Wald chi-square tests) revealed that, overall, the study area was not a significant factor (χ^2^ = 1.91; *p* = 0.39), while in interaction with vole abundance, it had an overall significant effect (χ^2^ = 9.01; *p* = 0.01). The second model revealed a positive effect of vole abundance in the previous season on the subsequent prevalence. Despite the significance of the main factor, the interaction of delayed abundance and study area was not significant (χ^2^ = 2.05; *p* = 0.36) as well as the effect of study area alone (χ^2^ = 2.93; *p* = 0.23). The impact of direct dependence on abundance varied spatially with Weissach showing a negative association, Jeeser a positive and Gotha showing no direct dependence on abundance (Figure 3a). For delayed abundance dependency of seroprevalence, no geographical pattern emerged (Figure 3b).

### 3.4. Detection of TULV RNA and Sequence Analysis

RT-PCR investigations were performed for lung samples from common and field voles originating from snap trapping and from voles found dead in live traps. Initially, lung samples from 333 common voles and 100 field voles from all four trapping areas were analyzed for TULV S segment-specific RNA (Table S2, Table S3). Common voles from all four trapping areas tested positive for TULV RNA. The mean RNA prevalence ranged between 7.3% and 27.4% (Table S4). TULV RNA was detected in common voles trapped during three consecutive years (2010–2012) in Jeeser, Gotha and Weissach. TULV RNA was only detected in seropositive field voles from Gotha (Table S1). In one field vole from Weissach a PUUV RNA sequence was detected, indicating a spillover infection [56].

Phylogenetic analysis of the S segment sequences revealed a typical clustering with similar sequences from geographically close trapping sites (Figure S2a). In addition, as recently defined [17], sequences from Jeeser and Gotha clustered within the Central North (CEN.N) clade and showed characteristic in-frame insertions of 3 nt (CAA; glutamine codon) in all obtained S segment sequences at position 790 (counting according to TULV S segment, accession number NC_005227). This finding was accompanied by a high pairwise sequence identity among representatives of the same clade (Table S5). TULV S segment sequences from Billerbeck and Weissach were members of the Central South (CEN.S) clade (Figure S2a; Table S5). In the Moravia prototype isolate (classified as EST.S; [17]) and sequences from Billerbeck and Weissach, the 3 nt insertion was missing. Analyses of partial L segment sequences showed the same patterns with sequences from Jeeser and Gotha in CEN.N clade, and sequences from Billerbeck and Weissach in the CEN.S clade (Figure S2b; see also Table S5). The M segment-based tree also showed the sequences from Jeeser and Gotha in CEN.N and sequences from Weissach in CEN.S; however, sequences from Billerbeck clustered here in the CEN.N clade (Figure S2c; see also Table S5). 

**Figure 4:**
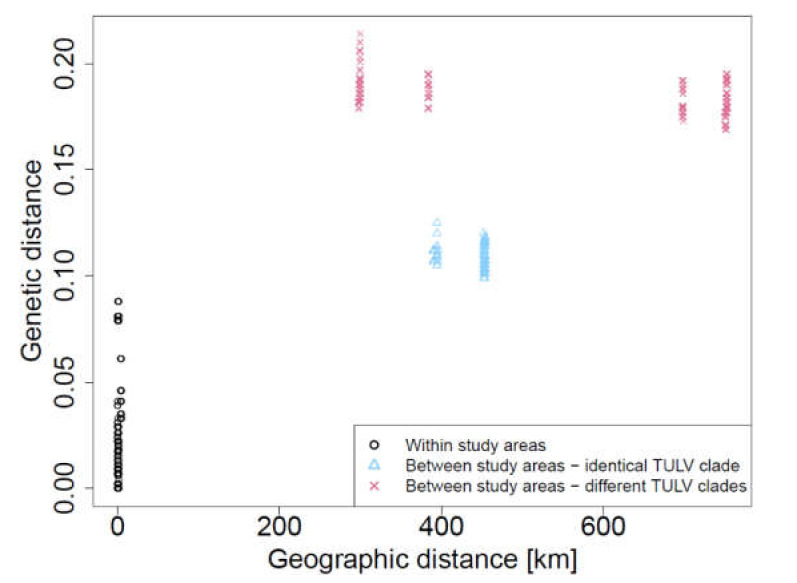
Isolation-by-distance relationship among TULV S segment sequences across the study areas in Germany. Red crosses represent data points for pairwise comparisons among the major phylogeographic clades TULV-CEN.S circulating in the study areas of Weissach and Billerbeck and TULV-CEN.N present in the study areas of Jeeser and Gotha.

**Figure 5:**
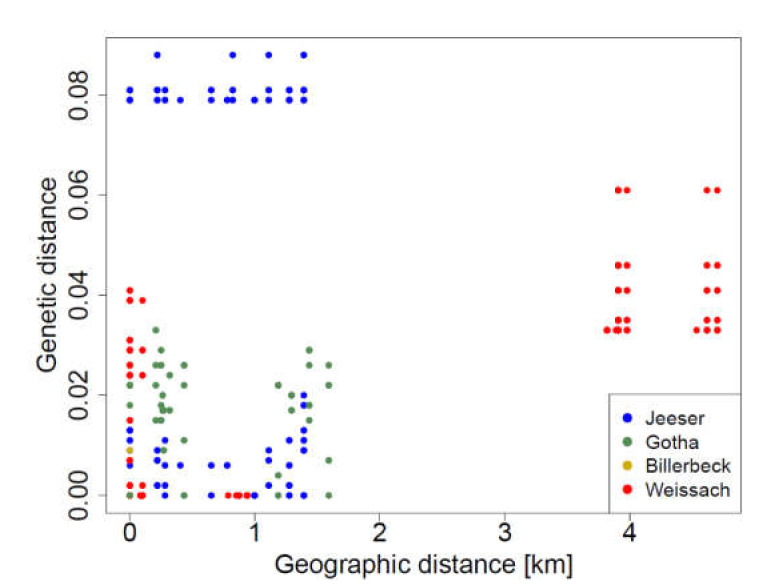
Relationships between TULV S segment sequences within the four study areas in Germany. Mantel tests detected significant isolation-by-distance patterns in the Weissach study area (red points; *p* < 0.0001) while there were no significant associations in the other sampling regions (all *p* > 0.2).

Sequence variation in TULV S segment followed a strong isolation-by-distance relationship across all studied areas in Germany (r^2^ = 0.619; Mantel test *p* < 0.0001; Figure 4). Consistent with larger geographic patterns of TULV variation [17], comparisons between study regions harboring different phylogenetic clades (TULV-CEN.S in the areas of Weissach and Billerbeck; TULV-CEN.N in the areas of Jeeser and Gotha) showed larger genetic divergence (*p*-distance: 18%–22%) than comparisons within TULV clades (*p*-distance: <13% between study areas). At the local scale, analysis revealed a highly significant isolation-by-distance pattern when all areas were tested jointly (r^2^ = 0.069; Mantel test *p* < 0.0001; Figure 5). Separate Mantel tests according to study area demonstrated that this was largely driven by data from Weissach with up to four kilometers distance between sampling sites (r^2^ = 0.576; *p* < 0.0001). Sequences from the other study areas with shorter maximum distances among sampling sites showed no significant isolation-by-distance patterns (Jeeser: r^2^ = 0.001; *p* = 0.274; Gotha: r^2^ = 0.005; *p* = 0.512; Billerbeck: *n* = 2 sequences, insufficient for statistical testing).

## 4. Discussion

The present study provides the first in depth account on spatial and temporal dynamics of TULV in relation to common vole population dynamics and their potential implications for molecular evolution in Central Europe. Conducting a multiannual monitoring field survey, which covered seasonal, annual and multi-annual fluctuations of common vole populations in four different regions, we were able to identify basal patterns of TULV dynamics within the rodent host populations in Germany. In comparison to PUUV, which was analyzed parallel to TULV in the same field survey (on additional forest plots; for details see [35,56]), TULV had a much broader geographical distribution (serological and RT-PCR detection in all four regions) and could be detected throughout Germany [9,57].

The estimated mean common vole abundance predominantly showed the typical seasonal fluctuations with lower numbers in spring, an increase during summer and a population peak in autumn (Figure 2; [26]). However, a few exceptions occurred in Weissach, in summer 2010 in Billerbeck and in summer 2012 in Gotha. Here, mean abundance peaked in summer. This deviation from the common seasonal pattern with autumn peaks could be due to small-scale processes. Common vole population dynamics are known to be influenced by various parameters such as predators and habitat factors [29] but also weather conditions [27,28]. At the small scale, dispersal capabilities of the common vole in relation to available nearby habitats can determine the local metapopulation structure [58]. These underlying, highly dynamic fluctuations may impact subsequent TULV dynamics at multiple scales.

The mean seroprevalences in common voles ranged in our study between 4.4% and 14.0%, with seasonal site-specific values between 0% and 28.0%. The range of the mean seroprevalences was similar to that observed in other studies in Germany (7.3%, [9], 16%, [6]), Austria (13.3%, [59]), France (7%, [60]), the Czech Republic (10%, [61], 9.7% [62]), Slovakia (6.6%, [63]), Belgium (7.7%, [64]) and Kazakhstan (15.6%, [65]). The mean RT-PCR detection rate in our study was at a similar level as the seroprevalences: it ranged between 7.3% and 27.4%, with seasonal site-specific values ranging between 0% and 37.5%. Results of previous studies revealed mean RNA detection rates of 15.6% [9] and 13.8% [57] in Germany and of 13.3% in Austria [59]. Similarly, a real-time RT-PCR-based study in the Netherlands indicated a TULV prevalence in the southern region of 41%, but of 12% to 45% in the northern regions [66]. The seasonal TULV RNA detection rate in another study in Central Germany reached 58.3% at one site in spring [57]. In contrast to this, TULV was detected only rarely in field voles, confirming again the major role of the common vole as the reservoir of TULV, and that field voles are mostly affected by spillover infections [9]. 

Sex was a determining factor for TULV dynamics on an individual level because males had an overall higher likelihood to be TULV seropositive. This is consistent with previous work on TULV [60] and can in part be explained by larger male home ranges and longer dispersal distances [58,67] increasing the chances of intraspecific contacts, and potentially leading to seroconversion. 

In contrast to our initial hypothesis, population-level TULV dynamics were not positively correlated to the current abundance. Our results suggest an overall positive delayed density dependence coupled with an overall negative direct density dependence (Table 2). This overall effect does appear to vary at lower spatial scales (interaction between abundance and site, Table 2). The generality of the assumption that high prevalence is always associated with high host abundance has been questioned repeatedly. Reil et al. [35], for example, found a strong seasonality in positive direct density dependence of PUUV. The latest results on PUUV in bank voles in Finland suggest that transient maternally derived immunity is a key feature of missing density dependence in populations [68]. For Sin nombre orthohantavirus (SNV) and its associated host, the deer mouse (*Peromyscus maniculatus*), a similar density dependence structure to the one presented here was described. Luis et al. [69] identified a strong delayed effect of deer mouse density on the prevalence of SNV. This is attributed to population fluctuations where the virus frequently becomes locally extinct due to missing host individuals. In such nonequilibrium, transient dynamics, peak host densities might not directly correspond to peak prevalence, as the virus survives at the metapopulation level rather than at a site-specific level. In these situations, immigration of nearby infected individuals is required, generating a time lag between the increase in host density and virus transmission at a particular site. Our data suggest that low winter survival in common vole populations with subsequent low spring abundances (Figure 2) could present such a bottleneck for site-specific TULV persistence. In this case, TULV might completely disappear from a plot and would need to be newly introduced by immigrating common voles from adjacent sources during the repopulation process [70,71]. Thereafter, it might take some time for the virus to spread within a newly established host population and, hence, the increase of TULV seroprevalence might be delayed in the following season. Given that the modern agricultural landscape supports a mosaic of suitable habitats for common voles, the degree of density dependence as well as the time lag is likely to vary between individual field sites depending on the distance to the nearest refuge as a source for recolonization to occur [72]. At a larger scale, this can be confirmed for TULV, as the different study areas varied in their expression of density-dependent patterns, likely reflecting differences in the landscape suitability structure and vole dynamics (Table 2). These results highlight that land-use patterns at the local and regional scale can have a large impact on the underlying pathogen dynamics and molecular evolution. Future work should therefore consider aspects of land use as explanatory variables for pathogen dynamics. However, our study had several limitations. Trapping could not be performed continuously at all sites in the last year of the study and the trap success and resulting lack of available sequences from the Billerbeck site might affect the large-scale applicability of the results. As this particular site was also characterized by a high prevalence of PUUV [56], the lack of samples limited the ability to investigate potential reassortment, though earlier publications using full genomes of TULV (and PUUV) or sequences from all genome segments have not provided evidence that reassortment is a common, or at least reasonably frequent, phenomenon in Central European phylogenetic clades and populations of these two orthohantavirus species [17,18,37,50].

Although we did not measure dispersal in the vole hosts directly, our molecular surveys conducted here indicate the buildup of isolation-by-distance patterns at the local scale, with sites closer together showing higher TULV relatedness compared to sites further apart. This can be interpreted as a host dispersal-driven metapopulation structure, where TULV is more likely to be shared between sites closer together. At larger geographical scales between study areas, genetic distances between TULV continue to increase (see also [50]). Isolation-by-distance relationships are not detectable for comparisons between sequences belonging to different TULV clades (Figure 4), which is consistent with long-term evolutionary divergence into functionally different “genotypes” within TULV in Germany [17,18]. The phylogenetic analyses of the partial S and L segment sequences from all four trapping sites confirmed the expected classification to the CEN.N clade (Jeeser) and CEN.S clade (Billerbeck). This classification is also indicated by an in-frame insertion/deletion of a glutamine codon sequence in the S segment. Surprisingly, the partial M segment sequences from Billerbeck clustered within the CEN.N clade. Sequence evolution in this part of the genome might be governed more strongly by the function of the glycoproteins encoded by the M segment and related differences in the selection pressure compared to the other segments [17,73]. It remains to be tested with larger datasets if a reassortment event in the evolutionary history of the Billerbeck TULV strains further contributed to the phylogenetic patterns. Reassortment events have been detected by in vitro studies of other hantaviruses resulting in the exchange of the M segment but leaving the S and L segments unaltered [74,75]. Reassortment events were also discussed as the reason for the evolution of different hantaviruses in nature (for review see [76]). 

## 5. Conclusions

This study focused on the temporal and spatial dynamics of multiannual common vole populations and highlighted determining factors. At the individual level, TULV infection risk was higher for males compared to females, likely reflecting different home ranges or aggressive interactions during the reproductive period. In contrast to our original hypothesis, TULV prevalence was negatively associated with current vole abundance, but positively dependent on the vole abundance of the previous season. This density dependence structure can be associated with transient, nonequilibrium host-pathogen dynamics, where frequent localized extinction events of hosts and pathogens (often during winter) on managed grasslands are followed by recolonization from nearby refuge areas. This observation is supported by isolation-by-distance patterns consistent with a dispersal-driven metapopulation structure at the local scale. However, the results are not consistent across all study sites, potentially reflecting different landscape structures mitigating the above-mentioned underlying mechanisms that lead to bottlenecks in local common vole populations.

## Figures and Tables

**Table 1:** TULV seroprevalence in common vole populations in four German areas from 2010 to 2013. Seroprevalence (%) in spring, summer and autumn of each year was estimated for three replicate grassland sites per area based on live and snap trapping. Values for the number of positive tested individuals (positive/total) per season are given for all sites in each study area. Percentages were calculated only for sites with ≥5 tested individuals (otherwise NA = not applicable).

			Weissach		Jeeser		Billerbeck		Gotha		
Year	Season	Site	Positive/ Total	%	Positive/ Total	%	Positive/ Total	%	Positive/ Total	%	Total %
2010	Spring	1	1/6	16.7	0/9	0	0/0	NA	0/1	NA	2.8
		2	0/0	NA	0/1	NA	0/0	NA	0/2	NA	
		3	0/5	0	0/3	NA	0/0	NA	0/9	0	
	Summer	1	6/47	12.8	1/12	8.3	0/0	NA	4/18	22	14.1
		2	3/18	16.7	6/24	25	0/1	NA	2/27	7.4	
		3	0/13	0	16/84	19	0/5	0	0/20	0	
	Autumn	1	0/0	NA	0/15	0	0/2	NA	2/35	5.7	6.8
		2	0/2	NA	3/22	14	2/18	11	1/41	2.4	
		3	4/17	23.5	5/30	17	0/0	NA	7/49	14	
2011	Spring	1	0/0	NA	0/0	NA	0/0	NA	2/6	33	12.5
		2	0/0	NA	0/0	NA	0/0	NA	1/16	6.3	
		3	0/0	NA	1/2	NA	0/1	NA	0/7	0	
	Summer	1	0/16	0	0/0	NA	0/0	NA	0/24	0	0.0
		2	0/17	0	0/0	NA	0/0	NA	0/11	0	
		3	0/103	0	0/0	NA	0/1	NA	0/14	0	
	Autumn	1	0/4	NA	1/10	10	0/0	NA	1/33	3	7.4
		2	0/0	NA	0/14	0	0/4	NA	3/60	5	
		3	12/110	10.9	0/4	NA	0/9	0	4/35	11	
2012	Spring	1	0/0	NA	0/5	0	0/2	NA	2/16	13	11.4
		2	0/0	NA	0/0	NA	0/2	NA	0/0	NA	
		3	0/0	NA	0/2	NA	0/0	NA	2/8	25	
	Summer	1	2/21	9.5	0/8	0	0/0	NA	2/29	6.9	9.1
		2	0/2	NA	0/0	NA	0/0	NA	2/14	14	
		3	0/0	NA	0/1	NA	0/0	NA	7/35	20	
	Autumn	1	0/0	NA	1/2	NA	No Trapping		0/30	0	12.0
		2	0/0	NA	0/0	NA			0/1	NA	
		3	0/3	NA	8/29	28			2/27	7.4	
2013	Spring	1	No Trapping		0/0	NA	No Trapping		0/0	NA	0.0
		2			0/0	NA			0/0	NA	
		3			0/0	NA			0/0	NA	
	Summer	1	No Trapping		0/2	NA	No Trapping		No Trapping		0.0
		2			0/0	NA					
		3			0/0	NA					
	Autumn	1	No Trapping		0/0	NA	No Trapping		0/1	NA	3.6
		2			0/1	NA			0/1	NA	
		3			1/21	4.8			0/4	NA	
Total			28/384	7.3	42/301	14	2/45	4.4	44/574	7.7	10.4

**Table 2:** Direct and delayed effects of common vole abundance (as index) in interaction with study area (SA) on TULV seroprevalence in the host population. The categorical factor contained three levels with Weissach as the reference category. Number of observations each = 43, degrees of freedom each = 6. Bold values indicate significance of *p* value (*p* < 0.05). SE = standard error; SD = standard deviation; z = Wald statistics defined as Estimate / SE.

	Same Season (Direct Effect)				Previous Season (Delayed Effect)			
Parameter	Estimate	SE	z	*p*	Estimate	SE	z	*p*
Intercept	−1.735	0.333	−5.215	0	−2.675	0.344	−7.785	0
Abundance	**−0.028**	**0.012**	**−2.335**	**0.02**	**0.023**	**0.01**	**2.315**	**0.021**
Jeeser	−0.481	0.45	−1.069	0.285	0.755	0.454	1.662	0.097
Gotha	−0.643	0.5	−1.286	0.198	0.261	0.49	0.531	0.595
Abundance: Jeeser	**0.055**	**0.018**	**3.001**	**0.003**	**−0.024**	**0.02**	**−1.212**	**0.226**
Abundance: Gotha	0.022	0.03	0.73	0.466	−0.033	0.036	−0.926	0.354
Random factor	Variance	SD			Variance	SD		
Site:SA	0	0			0.129	0.359		
SA	0	0			0	0		

## Data Availability

Relevant data are available upon request.

## Supplementary Materials

The following Supplementary Materials are available online at https://www.mdpi.com/article/10.3390/v13061132/s1, Figure S1: Phylogenetic tree of partial cytochrome *b* sequences of common voles from this study with reference sequences of the evolutionary lineages “Central”, “Eastern”, “Western” and “Italian”, and field vole (*Microtus agrestis*) and bank vole (*Myodes glareolus*) sequences as outgroup, Figure S2: Phylogenetic trees of partial S (a), L (b) and M (c) segment sequences of Tula orthohantavirus (TULV), Table S1: Number of trapped common voles per year, season and trapping methodology as well as derived abundance index as individuals (Ind.) per 100 trap nights (TN), Table S2: Results of TULV-IgG ELISA and RT-PCR investigations of field voles, Table S3: Accession numbers of cytochrome *b* gene sequences of common voles from the four regions in Germany, Table S4: Results of RT-PCR investigations of common voles, Table S5: Pairwise sequence similarities of TULV S, M and L segment sequences from the four trapping sites and of reference sequences of clades CEN.N and CEN.S, Table S6: Accession numbers of all common vole-derived Tula orthohantavirus (TULV) sequences used for consensus tree reconstruction (identical sequences are indicated).
