# Supplementary material for: Spatial and Temporal Dynamics and Molecular Evolution of Tula orthohantavirus in German Vole Populations
Source: Viruses. 2021 Jun 11;13(6):1132. doi: 10.3390/v13061132 (PMC8231151; doi:10.3390/v13061132)
Supplement: Supplementary file 1 [file viruses-13-01132-s001.zip › Figure S1-final.pptx]

## Slide 1
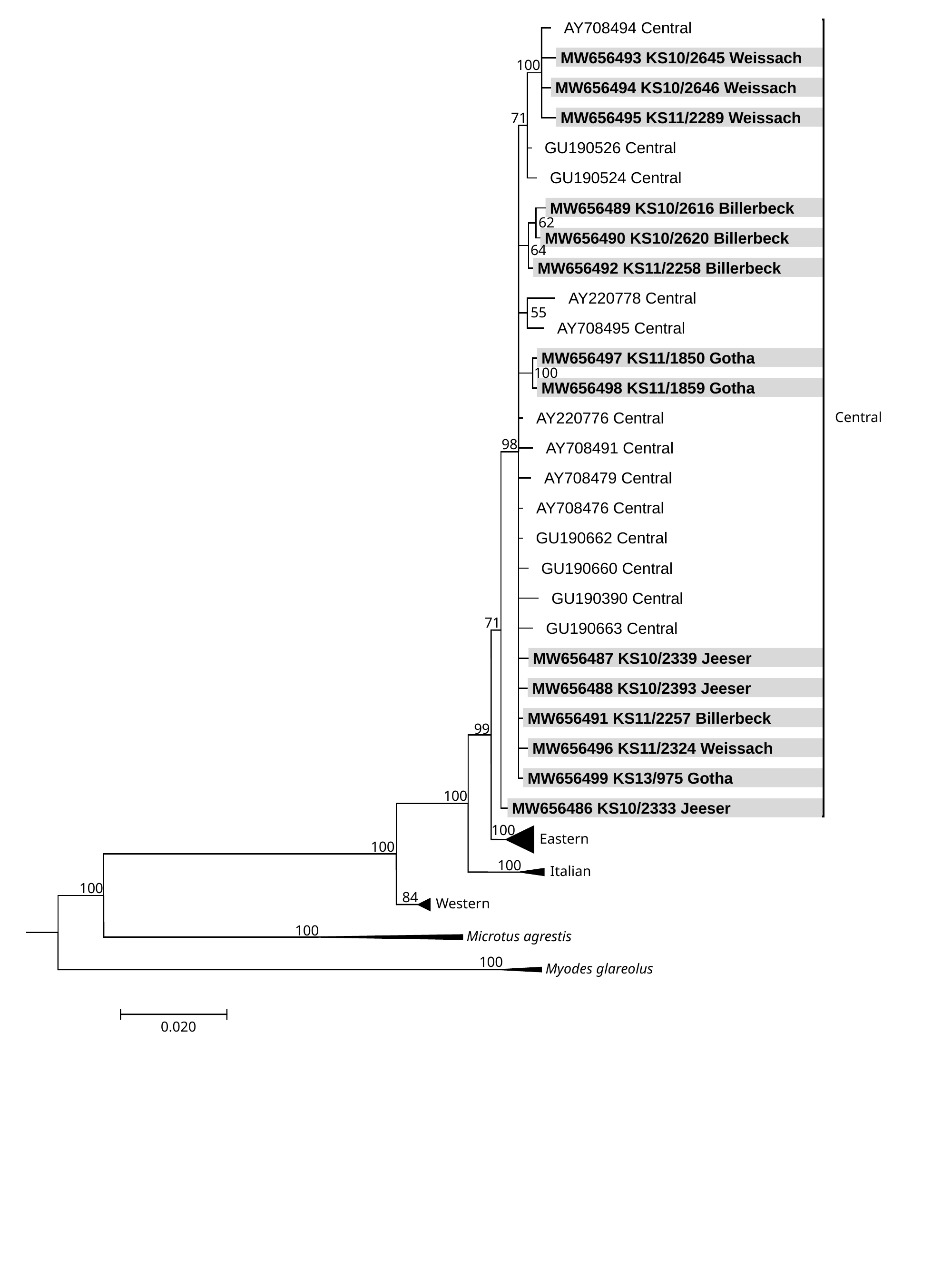

AY708494 Central
 MW656493 KS10/2645 Weissach
 MW656494 KS10/2646 Weissach
 MW656495 KS11/2289 Weissach
 GU190526 Central
 GU190524 Central
 MW656489 KS10/2616 Billerbeck
 MW656490 KS10/2620 Billerbeck
 MW656492 KS11/2258 Billerbeck
 AY220778 Central
 AY708495 Central
 MW656497 KS11/1850 Gotha
 MW656498 KS11/1859 Gotha
 AY220776 Central
Central
 AY708491 Central
 AY708479 Central
 AY708476 Central
 GU190662 Central
 GU190660 Central
 GU190390 Central
 GU190663 Central
 MW656487 KS10/2339 Jeeser
 MW656488 KS10/2393 Jeeser
 MW656491 KS11/2257 Billerbeck
 MW656496 KS11/2324 Weissach
 MW656499 KS13/975 Gotha
 MW656486 KS10/2333 Jeeser
 Eastern
 Italian
 Western
 Microtus agrestis
 Myodes glareolus
0.020
100
71
62
64
55
100
98
71
99
100
100
100
100
100
84
100
100
