# Supplementary material for: Spatial and Temporal Dynamics and Molecular Evolution of Tula orthohantavirus in German Vole Populations
Source: Viruses. 2021 Jun 11;13(6):1132. doi: 10.3390/v13061132 (PMC8231151; doi:10.3390/v13061132)
Supplement: Supplementary file 1 [file viruses-13-01132-s001.zip › Figure S2-final.pptx]

## Slide 1
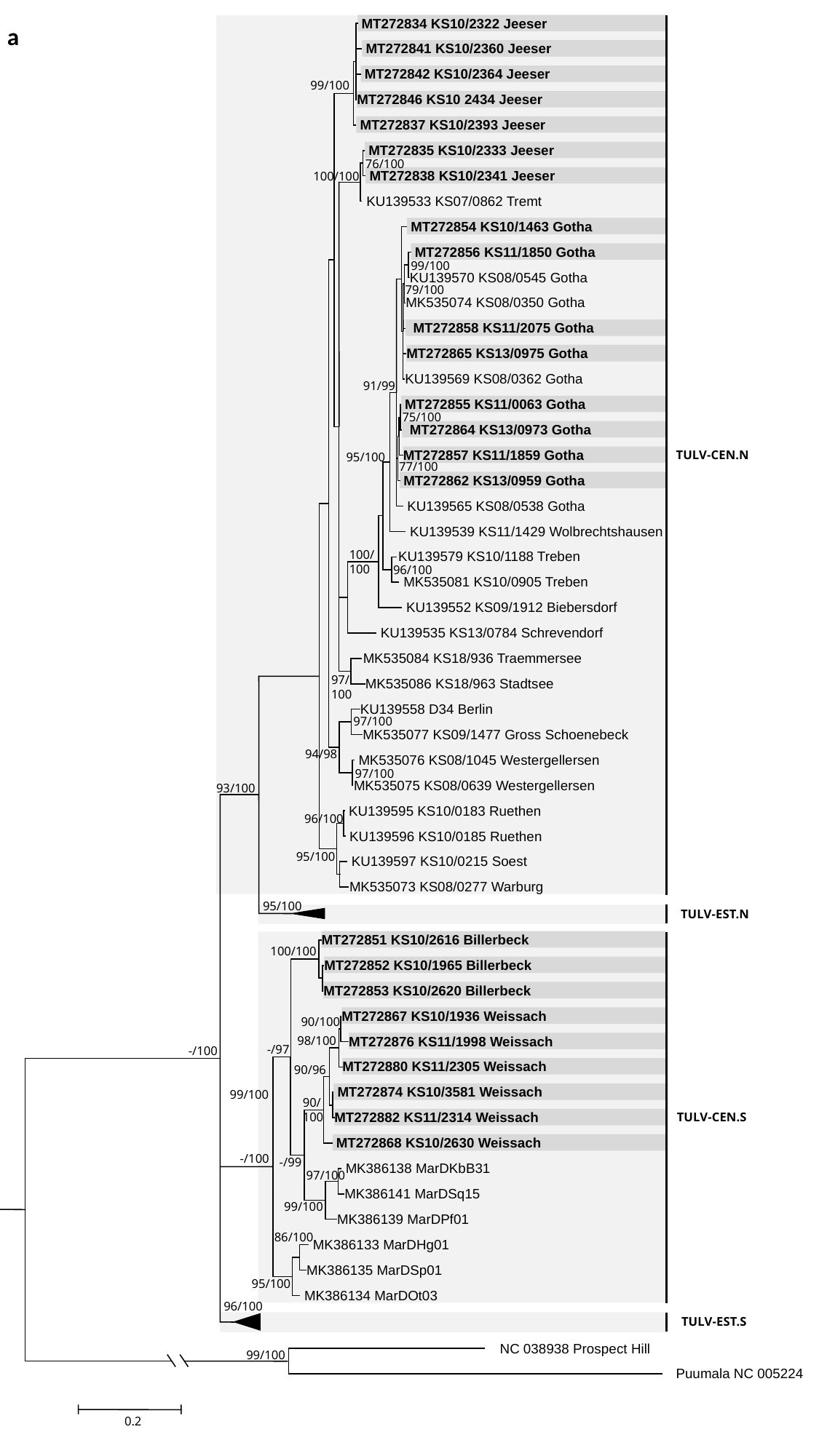

MT272834 KS10/2322 Jeeser
 MT272841 KS10/2360 Jeeser
 MT272842 KS10/2364 Jeeser
MT272846 KS10 2434 Jeeser
 MT272837 KS10/2393 Jeeser
 MT272835 KS10/2333 Jeeser
 MT272838 KS10/2341 Jeeser
 KU139533 KS07/0862 Tremt
 MT272854 KS10/1463 Gotha
 MT272856 KS11/1850 Gotha
KU139570 KS08/0545 Gotha
MK535074 KS08/0350 Gotha
 MT272858 KS11/2075 Gotha
MT272865 KS13/0975 Gotha
KU139569 KS08/0362 Gotha
 MT272855 KS11/0063 Gotha
 MT272864 KS13/0973 Gotha
MT272857 KS11/1859 Gotha
TULV-CEN.N
 MT272862 KS13/0959 Gotha
 KU139565 KS08/0538 Gotha
 KU139539 KS11/1429 Wolbrechtshausen
KU139579 KS10/1188 Treben
 MK535081 KS10/0905 Treben
 KU139552 KS09/1912 Biebersdorf
 KU139535 KS13/0784 Schrevendorf
MK535084 KS18/936 Traemmersee
MK535086 KS18/963 Stadtsee
KU139558 D34 Berlin
MK535077 KS09/1477 Gross Schoenebeck
 MK535076 KS08/1045 Westergellersen
MK535075 KS08/0639 Westergellersen
 KU139595 KS10/0183 Ruethen
 KU139596 KS10/0185 Ruethen
 KU139597 KS10/0215 Soest
MK535073 KS08/0277 Warburg
 TULV-EST.N
MT272851 KS10/2616 Billerbeck
MT272852 KS10/1965 Billerbeck
MT272853 KS10/2620 Billerbeck
MT272867 KS10/1936 Weissach
MT272876 KS11/1998 Weissach
MT272880 KS11/2305 Weissach
 MT272874 KS10/3581 Weissach
MT272882 KS11/2314 Weissach
TULV-CEN.S
 MT272868 KS10/2630 Weissach
 MK386138 MarDKbB31
MK386141 MarDSq15
MK386139 MarDPf01
 MK386133 MarDHg01
MK386135 MarDSp01
 MK386134 MarDOt03
 TULV-EST.S
 NC 038938 Prospect Hill
 Puumala NC 005224
0.2
a
99/100
76/100
100/100
99/100
79/100
91/99
75/100
95/100
77/100
100/
100
96/100
97/
100
97/100
94/98
97/100
93/100
96/100
95/100
95/100
100/100
90/100
98/100
-/97
-/100
90/96
99/100
90/
100
-/100
-/99
97/100
99/100
86/100
95/100
96/100
99/100

## Slide 2
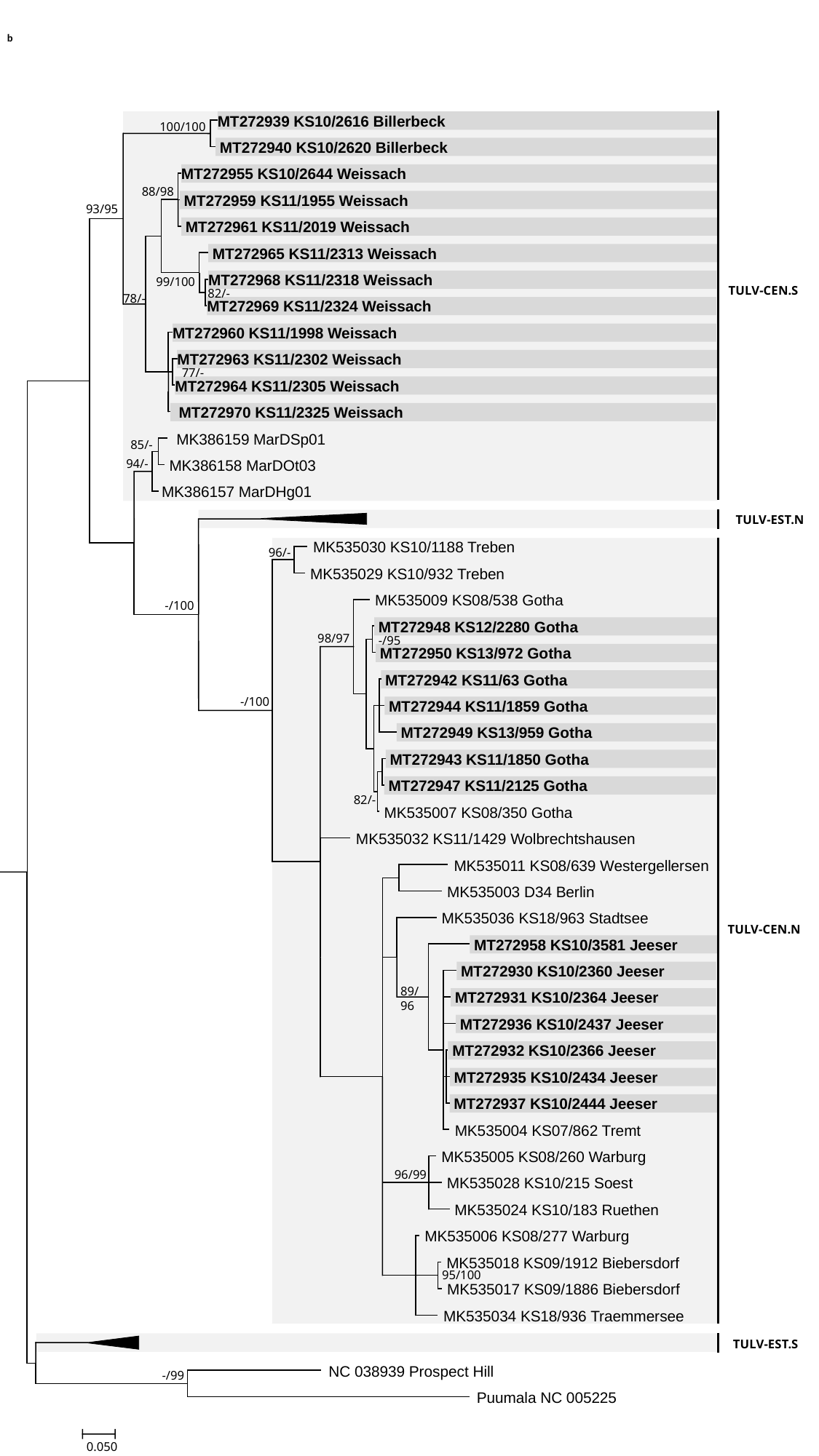

# b
MT272939 KS10/2616 Billerbeck
 MT272940 KS10/2620 Billerbeck
MT272955 KS10/2644 Weissach
 MT272959 KS11/1955 Weissach
 MT272961 KS11/2019 Weissach
 MT272965 KS11/2313 Weissach
MT272968 KS11/2318 Weissach
TULV-CEN.S
MT272969 KS11/2324 Weissach
MT272960 KS11/1998 Weissach
MT272963 KS11/2302 Weissach
MT272964 KS11/2305 Weissach
 MT272970 KS11/2325 Weissach
 MK386159 MarDSp01
 MK386158 MarDOt03
MK386157 MarDHg01
 TULV-EST.N
 MK535030 KS10/1188 Treben
 MK535029 KS10/932 Treben
 MK535009 KS08/538 Gotha
 MT272948 KS12/2280 Gotha
 MT272950 KS13/972 Gotha
 MT272942 KS11/63 Gotha
 MT272944 KS11/1859 Gotha
 MT272949 KS13/959 Gotha
 MT272943 KS11/1850 Gotha
 MT272947 KS11/2125 Gotha
 MK535007 KS08/350 Gotha
 MK535032 KS11/1429 Wolbrechtshausen
 MK535011 KS08/639 Westergellersen
 MK535003 D34 Berlin
 MK535036 KS18/963 Stadtsee
TULV-CEN.N
 MT272958 KS10/3581 Jeeser
 MT272930 KS10/2360 Jeeser
 MT272931 KS10/2364 Jeeser
 MT272936 KS10/2437 Jeeser
 MT272932 KS10/2366 Jeeser
 MT272935 KS10/2434 Jeeser
 MT272937 KS10/2444 Jeeser
 MK535004 KS07/862 Tremt
 MK535005 KS08/260 Warburg
 MK535028 KS10/215 Soest
 MK535024 KS10/183 Ruethen
 MK535006 KS08/277 Warburg
 MK535018 KS09/1912 Biebersdorf
 MK535017 KS09/1886 Biebersdorf
 MK535034 KS18/936 Traemmersee
 TULV-EST.S
 NC 038939 Prospect Hill
 Puumala NC 005225
0.050
100/100
88/98
93/95
99/100
82/-
78/-
77/-
85/-
94/-
96/-
-/100
98/97
-/95
-/100
82/-
89/
96
96/99
95/100
-/99

## Slide 3
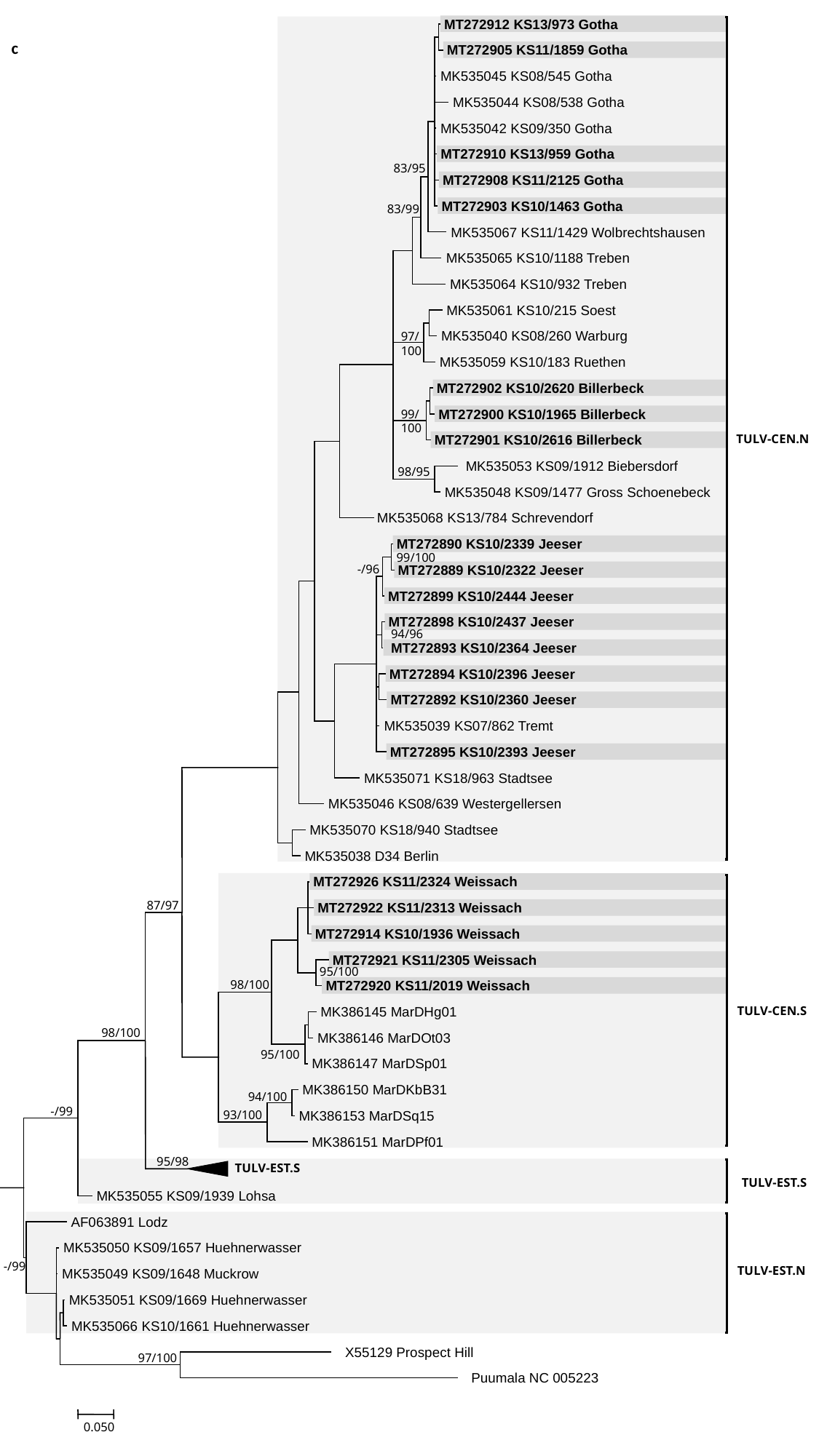

MT272912 KS13/973 Gotha
 MT272905 KS11/1859 Gotha
 MK535045 KS08/545 Gotha
 MK535044 KS08/538 Gotha
 MK535042 KS09/350 Gotha
 MT272910 KS13/959 Gotha
 MT272908 KS11/2125 Gotha
 MT272903 KS10/1463 Gotha
 MK535067 KS11/1429 Wolbrechtshausen
 MK535065 KS10/1188 Treben
 MK535064 KS10/932 Treben
 MK535061 KS10/215 Soest
 MK535040 KS08/260 Warburg
 MK535059 KS10/183 Ruethen
 MT272902 KS10/2620 Billerbeck
 MT272900 KS10/1965 Billerbeck
 MT272901 KS10/2616 Billerbeck
TULV-CEN.N
 MK535053 KS09/1912 Biebersdorf
 MK535048 KS09/1477 Gross Schoenebeck
 MK535068 KS13/784 Schrevendorf
 MT272890 KS10/2339 Jeeser
 MT272889 KS10/2322 Jeeser
 MT272899 KS10/2444 Jeeser
 MT272898 KS10/2437 Jeeser
 MT272893 KS10/2364 Jeeser
 MT272894 KS10/2396 Jeeser
 MT272892 KS10/2360 Jeeser
 MK535039 KS07/862 Tremt
 MT272895 KS10/2393 Jeeser
 MK535071 KS18/963 Stadtsee
 MK535046 KS08/639 Westergellersen
 MK535070 KS18/940 Stadtsee
 MK535038 D34 Berlin
 MT272926 KS11/2324 Weissach
 MT272922 KS11/2313 Weissach
 MT272914 KS10/1936 Weissach
 MT272921 KS11/2305 Weissach
 MT272920 KS11/2019 Weissach
 MK386145 MarDHg01
TULV-CEN.S
 MK386146 MarDOt03
 MK386147 MarDSp01
 MK386150 MarDKbB31
 MK386153 MarDSq15
 MK386151 MarDPf01
 TULV-EST.S
 MK535055 KS09/1939 Lohsa
 AF063891 Lodz
 MK535050 KS09/1657 Huehnerwasser
TULV-EST.N
 MK535049 KS09/1648 Muckrow
 MK535051 KS09/1669 Huehnerwasser
 MK535066 KS10/1661 Huehnerwasser
 X55129 Prospect Hill
 Puumala NC 005223
0.050
# c
83/95
83/99
97/
100
99/
100
98/95
99/100
-/96
94/96
87/97
95/100
98/100
98/100
95/100
94/100
-/99
93/100
95/98
 TULV-EST.S
-/99
97/100
