# Supplementary material for: Spatial and Temporal Dynamics and Molecular Evolution of Tula orthohantavirus in German Vole Populations
Source: Viruses. 2021 Jun 11;13(6):1132. doi: 10.3390/v13061132 (PMC8231151; doi:10.3390/v13061132)
Supplement: Supplementary file 1 [file viruses-13-01132-s001.zip › Supplementary Figure S1 and S2 legends.pdf]

Figure S1. Phylogenetic tree of partial cytochrome *b* sequences of common voles from this study with reference sequences of the evolutionary lineages "Central", "Eastern", "Western" and "Italian", and field vole (*Microtus agrestis*) and bank vole (*Myodes glareolus*) sequences as outgroup. The Bayesian tree is based on 4 x 10^6 generations and a burn-in phase of 25% using MrBayes v3.2.6 [1]. The most suitable substitution model, the Hasegawa-Kishino-Yano model with invariant sites and a gamma distributed shape parameter (HKY+I+G), was obtained with the aid of jModelTest v2.1.6 [2]. All tree reconstructions were done on CIPRES Science Gateway [3]. Algorithm: Markov chain Monte Carlo, Substitution model: HKY+I+G, Alignment length: 825 nt.

Figure S2. Phylogenetic trees of partial S (a), L (b) and M (c) segment sequences of Tula orthohantavirus (TULV). (a) Consensus phylogenetic tree of the partial S segment sequences of TULV (alignment length 549 nucleotides (nt), positions 406-951, counting according to TULV S segment, accession number NC_005227). (b) Consensus phylogenetic tree of the partial L segment sequences of TULV (alignment length 327 nucleotides (nt), positions 2983-3309, counting according to TULV L segment, accession number NC_005226). (c) Consensus phylogenetic tree of the partial M segment sequences of TULV (alignment length 350 nucleotides (nt), positions 2535-2884, counting according to TULV M segment, accession number NC_005228). The consensus trees are based on Bayesian analyses with 10^7 generations and a burn-in phase of 25%, and Maximum-Likelihood analyses, with 1,000 bootstraps and 50% cut-off using the General Time Reversible (GTR) substitution model with invariant sites and a gamma distributed shape parameter for both algorithms. Bootstrap values > 75% are given before the slash and posterior probabilities > 95 % from Bayesian analyses are given after the slash for major nodes supported by both algorithms. The tree reconstructions were done via CIPRES [3]. Alignments were constructed under Bioedit (V7.2.3.) [4] using the Clustal W Multiple Alignment algorithm implemented in the program. Names in bold and highlighted with a grey layer indicate newly generated sequences (see Table S5 and Table S6). Identical sequences are listed in Table S5. Clade designations were made according to Saxenhofer et. al. [5]. Compressed branches, indicated by triangles, include the following sequences TULV-EST.S (S: MK386130-MK386132, MK386136, MK386137, MK386140, Z69991; M: MK386142- MK386144, MK386148, MK386149, MK386152; MK535055, Z69993; L: MK386154- MK386156, MK386160, MK386161, MK386164) and TULV-EST.N (S: AF063892, KU139555, KU139563, MK535078, MK535083; M: AF063891, MK535049- MK535051, MK535066).

References

1. Ronquist, F.; Teslenko, M.; van der Mark, P.; Ayres, D.L.; Darling, A.; Höhna, S.; Larget, B.; Liu, L.; Suchard, M.A.; Huelsenbeck, J.P. MrBayes 3.2: efficient Bayesian phylogenetic inference and model choice across a large model space. *Syst. Biol.* **2012**, *61*, 539-542.
2. Darriba, D.; Taboada, G.L.; Doallo, R.; Posada, D. jModelTest 2: more models, new heuristics and parallel computing. *Nat. Methods* **2012**, *9*, 772.
3. Miller, M.A.; Pfeiffer, W.; Schwartz, T. The CIPRES science gateway: A community resource for phylogenetic analyses. In *Extreme Digital Discovery, Proceedings of the 2011 TeraGrid Conference, Salt Lake City, UT, USA, 18–21 July 2011*; Association for Computing Machinery: New York, NY, USA, 2011; pp. 1–8.4.
Hall, T. BioEdit: An Important Software for Molecular Biology. *GERF Bull. Biosci.* **2011**, *2*, 60–61.
5. Saxenhofer, M.; Schmidt, S.; Ulrich, R.G.; Heckel, G. Secondary Contact between Diverged Host Lineages Entails Ecological Speciation in a European Hantavirus. *PLoS Biol.* **2019**, *17*, e3000142.
