# Supplementary material for: Spatial and Temporal Dynamics and Molecular Evolution of Tula orthohantavirus in German Vole Populations
Source: Viruses. 2021 Jun 11;13(6):1132. doi: 10.3390/v13061132 (PMC8231151; doi:10.3390/v13061132)
Supplement: Supplementary file 1 [file viruses-13-01132-s001.zip › Supplementary Tables for conversion.pdf]

Table S1. Number of trapped common voles per year, season and trapping methodology as well as derived abundance index as individuals (Ind.) per 100 trap nights (TN). Total trapping effort per trapping session and site was 294 traps (49 snap traps (ST) and 49 live traps (LT) for 3 days).

			Weissach			Jeeser			Billerbeck			Gotha		
**Year**	**Season**	**Site**	**N LT**	**N ST**	**Abundance [Ind./ 100TN]**	**N LT**	**N ST**	**Abundance [Ind./ 100TN]**	**N LT**	**N ST**	**Abundance [Ind./ 100TN]**	**N LT**	**N ST**	**Abundance [Ind./ 100TN]**
		1	0	6	2.0	12	0	4.1	1	0	0.3	1	0	0.3
	Spring	2	2	0	0.7	3	0	1.0	0	0	0.0	0	2	0.7
		3	0	5	1.1	3	0	1.0	0	0	0.0	8	1	3.1
		1	35	14	16.7	4	4	2.7	1	0	0.3	18	0	6.1
2010	Summer	2	13	5	6.1	15	10	8.5	2	3	1.7	25	2	9.2
		3	30	11	13.9	85	10	32.3	0	0	0.0	19	1	6.8
		1	0	0	0.0	14	1	5.1	17	7	8.2	38	2	13.6
	Autumn	2	1	1	0.7	23	1	8.2	1	0	0.3	43	12	18.7
		3	17	2	6.5	29	3	10.9	2	0	0.7	40	11	17.3
		1	0	0	0.0	0	0	0.0	0	0	0.0	5	0	1.7
	Spring	2	0	0	0.0	0	0	0.0	1	0	0.3	15	2	5.8
		3	0	0	0.0	2	0	0.7	0	0	0.0	7	0	2.4
		1	11	4	5.1	0	0	0.0	0	0	0.0	22	5	9.2
2011	Summer	2	22	0	7.5	0	0	0.0	0	1	0.3	8	2	3.4
		3	116	20	46.3	0	1	0.3	0	0	0.0	12	5	5.8
		1	3	1	1.4	8	3	3.7	3	1	1.4	28	6	11.6
	Autumn	2	0	0	0.0	15	0	5.1	5	4	3.1	57	7	21.8
		3	78	36	38.8	5	0	1.7	0	0	0.0	29	8	12.6
		1	0	0	0.0	5	0	1.7	1	1	0.7	16	2	6.1
	Spring	2	0	0	0.0	0	0	0.0	0	0	0.0	0	0	0.0
		3	0	0	0.0	2	0	0.7	0	2	0.7	6	2	2.7
		1	20	5	8.5	8	0	2.7	0	0	0.0	33	0	11.2
2012	Summer	2	1	1	0.7	0	0	0.0	0	0	0.0	12	3	5.1
		3	0	0	0.0	0	1	0.3	0	0	0.0	36	4	13.6
		1	0	0	0.0	3	0	1.0				33	7	13.6
	Autumn	2	0	0	0.0	0	0	0.0		No Trapping		0	0	0.0
		3	3	0	1.0	29	3	10.9				33	5	12.9
		1				0	0	0.0				0	0	0.0
	Spring	2		No Trapping		0	0	0.0		No Trapping		0	0	0.0
		3				0	0	0.0				0	0	0.0
		1				3	5	2.7						
2013	Summer	2		No Trapping		0	0	0.0		No Trapping			No Trapping	
		3				0	0	0.0						
		1				0	0	0.0				1	0	0.3
	Autumn	2		No Trapping		1	0	0.3		No Trapping		0	1	0.3
		3				21	0	7.1				3	1	1.4
	Total		352	111		290	42		34	19		548	91	

Table S2. Results of TULV-IgG ELISA and RT-PCR investigations of field voles

Year	Season	Weissach			Jeeser			Billerbeck			Gotha		
		N individuals trapped	ELISA pos/total	RT-PCR pos/total	N individuals trapped	ELISA pos/total	RT-PCR pos/total	N individuals trapped	ELISA pos/total	RT-PCR pos/total	N individuals trapped	ELISA pos/total	RT-PCR pos/total
2010	Spring	4	2/4	0/4	9	0/6	0/1	0	NA	NA	0	NA	NA
	Summer	2	1*/2	1**/2	7	0/7	0/6	0	NA	NA	14	0/12	0/9
	Autumn	0	NA	NA	1	0/1	0/1	0	NA	NA	36	1/24	0/12
2011	Spring	0	NA	NA	2	0/2	0/0	0	NA	NA	4	0/4	0/2
	Summer	1	0/1	0/1	1	0/0	0/0	0	NA	NA	17	0/15	0/9
	Autumn	1	0/1	0/0	6	0/6	0/3	1	0/1	0/1	16	0/15	0/6
2012	Spring	1	0/1	0/0	1	0/0	0/0	0	NA	NA	1	0/1	0/0
	Summer	0	NA	NA	2	0/2	0/2	No trapping			15	2/15	1/14
	Autumn	0	NA	NA	8	2/6	0/1	No trapping			20	4/19	3/16
2013	Spring	No trapping			0	NA	NA	No trapping			0	NA	NA
	Summer	No trapping			6	0/6	0/6	No trapping			0	NA	NA
	Autumn	No trapping			0	0/0	0/0	No trapping			4	1/4	1/4
Total		9	3**/9	1*/7	43	2/36	0/20	1	0/1	0/1	127	8/109	5/72

N, number; NA, not applicable; pos, positive

* tested positive in PUUV and TULV IgG ELISA; ** sequencing revealed a Puumala orthohantavirus sequence, explained by spillover infection (Binder et al., 2020).

Table S3. Accession numbers of cytochrome *b* gene sequences of common voles from the four regions in Germany

ID	Trapping location	Collection date	Accession number
KS10/2333	Jeeser	28.07.2010	MW656486
KS10/2339	Jeeser	27.07.2010	MW656487
KS10/2393	Jeeser	28.07.2010	MW656488
KS10/2616	Billerbeck	01.10.2010	MW656489
KS10/2620	Billerbeck	01.10.2010	MW656490
KS11/2257	Billerbeck	11.10.2011	MW656491
KS11/2258	Billerbeck	11.10.2011	MW656492
KS10/2645	Weissach	27.08.2010	MW656493
KS10/2646	Weissach	27.08.2010	MW656494
KS11/2289	Weissach	21.10.2011	MW656495
KS11/2324	Weissach	25.10.2011	MW656496
KS11/1850	Gotha	16.07.2011	MW656497
KS11/1859	Gotha	14.07.2011	MW656498
KS13/0975	Gotha	26.09.2012	MW656499

Table S4. Results of RT-PCR investigations of common voles

		Weissach		Jeeser		Billerbeck		Gotha	
Year	Season	pos/total	%	pos/total	%	pos/total	%	pos/total	%
2010	Spring	0/11	0	0/0	NA	0/0	NA	1/7	14.3
	Summer	9/28	32.1	15/40	37.5	0/3	NA	1/19	5.3
	Autumn	1/3	NA	2/12	16.7	2/6	33.3	1/26	3.8
2011	Spring	0/0	NA	0/0	NA	0/0	NA	0/2	NA
	Summer	3/40	12	0/0	NA	0/1	NA	2/17	11.8
	Autumn	9/40	25	0/4	NA	0/5	0	3/28	10.7
2012	Spring	0/0	NA	0/0	NA	0/3	NA	0/4	NA
	Summer	2/8	33.3	0/1	NA	No trapping		0/9	0
	Autumn	0/0	NA	0/3	NA	No trapping		1/9	11.1
2013	Spring	No trapping		0/0	NA	No trapping		0/0	NA
	Summer	No trapping		0/0	NA	No trapping		0/0	NA
	Autumn	No trapping		0/2	NA	No trapping		0/2	NA
Total		24/130	22	17/62	27.4	2/18	11.1	9/123	7.3

NA, not applicable; pos, positive

Table S5. Pairwise sequence similarities of TULV S, M and L segment sequences from the four trapping sites and of reference sequences of clades CEN.N and CEN.S.

Trapping site	Sequence identity within trapping site	Similarity with reference CEN.N KS08/0350 Gotha	Similarity with reference CEN.N KS07/0862 Tremt	Similarity with reference CEN.S MarDSp01
Jeeser	S: 0.912-1<br>M: 0.962-1<br>L: 0.917-1	S: 0.888-0.896<br>M: 0.850-0.870<br>L: 0.853-0.871	S: 0.916-0.992<br>M: 0.865-0.985<br>L: 0.923-0.987	S: 0.794-0.806<br>M: 0.816-0.830<br>L: 0.795-0.828
Gotha	S: 0.967-1<br>M: 0.982-1<br>L: 0.958-1	S: 0.978-0.994<br>M: 0.985-0.997<br>L: 0.963-0.963	S: 0.888-0.901<br>M: 0.859-0.867<br>L: 0.840-0.868	S: 0.803-0.806<br>M: 0.841-0.847<br>L: 0.813-0.825
Billerbeck	S: 0.989-0.998<br>M: 0.983-0.993<br>L: 0.978-0.978	S: 0.803-0.808<br>M: 0.905-0.910<br>L: 0.795-0.798	S: 0.801-0.805<br>M: 0.839-0.850<br>L: 0.788-0.792	S: 0.880-0.884<br>M: 0.841-0.841<br>L: 0.847-0.850
Weissach	S: 0.939-1<br>M: 0.953-1<br>L: 0.917-1	S: 0.786-0.814<br>M: 0.804-0.824<br>L: 0.801-0.834	S: 0.801-0.825<br>M: 0.807-0.824<br>L: 0.785-0.819	S: 0.871-0.886<br>M: 0.893-0.916<br>L: 0.859-0.877
All other CEN.N sequences	n.d.	S: 0.872-0.990<br>M: 0.853-0.997<br>L: 0.847-0.954	S: 0.883-0.927<br>M: 0.841-0.905<br>L: 0.834-0.896	S: 0.792-0.816<br>M: 0.810-0.847<br>L: 0.798-0.862
All other CEN.S sequences	n.d.	S: 0.794-0.817<br>M: 0.836-0.844<br>L: 0.810-0.819	S: 0.785-0.805<br>M: 0.821-0.830<br>L: 0.810-0.840	S: 0.879-0.970<br>M: 0.982-0.988<br>L: 0.837-0.981

Sequence length: S (549 nt for Jeeser and Gotha, 546 nt for Billerbeck and Weissach), M 618 nt and L 411 nt. Comparison with reference sequences S (549 nt for CEN.N sequences, 546 nt for CEN.S sequences), M 348 nt and L 327 nt. All other CEN.N and CEN.S sequences are given in Figure S2.

n.d., not done.

Table S6. Accession numbers of all common vole-derived Tula orthohantavirus (TULV) sequences used for consensus tree reconstruction (identical sequences are indicated).

Isolate	Trapping location	Host	Collection date	S	M	L
KS10/2322	Jeeser	*Microtus arvalis*	2010 summer	MT272834 (identical to MT272836, MT272840, MT272848)	MT272889	MT272927
KS10/2333	Jeeser	*Microtus arvalis*	2010 summer	MT272835 (identical to MT272843)	-	
KS10/2339	Jeeser	*Microtus arvalis*	2010 summer	MT272836 (identical to MT272834, MT272840, MT272848)	MT272890	MT272928
KS10/2341	Jeeser	*Microtus arvalis*	2010 summer	MT272838 (identical to MT272844, MT272845, MT272839)		
KS10/2346	Jeeser	*Microtus arvalis*	2010 summer	MT272839 (identical to MT272844, MT272845, MT272838)		
KS10/2357	Jeeser	*Microtus arvalis*	2010 summer	MT272840 (identical to MT272834, MT272836, MT272848)	MT272891 (identical to MT272897, MT272899, MT272894)	MT272929 (identical to MT272932)
KS10/2360	Jeeser	*Microtus arvalis*	2010 summer	MT272841	MT272892	MT272930
KS10/2364	Jeeser	*Microtus arvalis*	2010 summer	MT272842	MT272893	MT272931
KS10/2366	Jeeser	*Microtus arvalis*	2010 summer	MT272843 (identical to MT272835)	MT272894 (identical to MT272897, MT272891, MT272899)	MT272932 (identical to MT272929)

Table S6 (continued)

Isolate	Trapping location	Host	Collection date	S	M	L
KS10/2393	Jeeser	*Microtus arvalis*	2010 summer	MT272837 (identical to MT272849)	MT272895	MT272933
KS10/2396	Jeeser	*Microtus arvalis*	2010 summer	MT272844 (identical to MT272838, MT272845, MT272839)	MT272896	MT272934
KS10/2400	Jeeser	*Microtus arvalis*	2010 summer	MT272845 (identical to MT272844, MT272838, MT272839)		
KS10/2434	Jeeser	*Microtus arvalis*	2010 summer	MT272846 (identical to MT272847, MT272850)	MT272897 (identical to MT272899, MT272891, MT272894)	MT272935
KS10/2437	Jeeser	*Microtus arvalis*	2010 summer	MT272847 (identical to MT272846, MT272850)	MT272898	MT272936
KS10/2444	Jeeser	*Microtus arvalis*	2010 summer	MT272848 (identical to MT272836, MT272840, MT272834)	MT272899 (identical to MT272897, MT272891, MT272894)	MT272937
KS10/3631	Jeeser	*Microtus arvalis*	2010 fall	MT272849 (identical to MT272837)		MT272958
KS11/0016	Jeeser	*Microtus arvalis*	2010 fall	MT272850 (identical to MT272846, MT272847)		
KS10/1965	Billerbeck	*Microtus arvalis*	2010 summer	MT272852	MT272900	MT272939
KS10/2616	Billerbeck	*Microtus arvalis*	2010 fall	MT272851	MT272901	MT272940
KS10/2620	Billerbeck	*Microtus arvalis*	2010 fall	MT272853	MT272902	

Table S6 (continued)

Isolate	Trapping location	Host	Collection date	S	M	L
KS10/1463	Gotha	*Microtus arvalis*	2010 spring	MT272854 (identical to MT272861, MT272863, MT272866)	MT272903 (identical to MT272913 MT272911)	MT272941 (identical to MT272948)
KS11/0063	Gotha	*Microtus arvalis*	2010 fall	MT272855 (identical to MT272859)		MT272942 (identical to MT272946)
KS11/1850	Gotha	*Microtus arvalis*	2011 summer	MT272856	MT272904	MT272943
KS11/1859	Gotha	*Microtus arvalis*	2011 summer	MT272857	MT272905	MT272944
KS11/2075	Gotha	*Microtus arvalis*	2011 fall	MT272858 (identical to MT272860)	MT272906 (identical to MT272908)	MT272945 (identical to MT272947)
KS11/2084	Gotha	*Microtus arvalis*	2011 fall	MT272859 (identical to MT272855)	MT272907	MT272946 (identical to MT272942)
KS11/2125	Gotha	*Microtus arvalis*	2011 fall	MT272860 (identical to MT272858)	MT272908 (identical to MT272906)	MT272947 (identical to MT272945)
KS12/2280	Gotha	*Microtus arvalis*	2012 summer	MT272861 (identical to MT272854, MT272863, MT272866)	MT272909 (identical to MT272911, MT272913, MT272903)	MT272948 (identical to MT272941)
KS13/0959	Gotha	*Microtus arvalis*	2013 fall	MT272862	MT272910	MT272949
KS13/0972	Gotha	*Microtus arvalis*	2013 fall	MT272863 (identical to MT272861, MT272854, MT272866)	MT272911 (identical to MT272913,	MT272950

Table S6 (continued)

Isolate	Trapping location	Host	Collection date	S	M	L
KS13/0972	Gotha	*Microtus arvalis*	2013 fall		MT272909, MT272903)	
KS13/0973	Gotha	*Microtus arvalis*	2013 fall	MT272864	MT272912 (identical to MT272906)	MT272951
KS13/0975	Gotha	*Microtus arvalis*	2013 fall	MT272865		
KS13/0978	Gotha	*Microtus arvalis*	2013 fall	MT272866 (identical to MT272861, MT272863, MT272854)	MT272913 (identical to MT272911, MT272909, MT272903)	
KS10/1936	Weissach	*Microtus arvalis*	2010 summer	MT272867 (identical to MT272879)	MT272914	MT272952 (identical to MT272960)
KS10/2630	Weissach	*Microtus arvalis*	2010 fall	MT272868 (identical to MT272869, MT272870, MT272871, MT272872,		MT272953 (identical to MT272959,
KS10/2634	Weissach	*Microtus arvalis*	2010 fall	MT272869 (identical to MT272868, MT272870, MT272871, MT272872, MT272875, MT272877, MT272886, MT272888, MT272873)	MT272915 (identical to MT272920, MT272916, MT272917, MT272918, MT272919)	MT272954

Table S6 (continued)

Isolate	Trapping location	Host	Collection date	S	M	L
KS10/2636	Weissach	*Microtus arvalis*	2010 summer	MT272870 (identical to MT272869, MT272868, MT272871, MT272872, MT272875, MT272877, MT272886, MT272888, MT272873)	MT272916 (identical to MT272915, MT272920, MT272917, MT272918, MT272919)	
KS10/2644	Weissach	*Microtus arvalis*	2010 summer	MT272871 (identical to MT272869, MT272870, MT272868, MT272872, MT272875, MT272877, MT272886, MT272888, MT272873)	MT272917 (identical to MT272915, MT272916, MT272920, MT272918, MT272919)	MT272955
KS10/2645	Weissach	*Microtus arvalis*	2010 summer	MT272872 (identical to MT272869, MT272870, MT272871, MT272868, MT272875, MT272877, MT272886, MT272888, MT272873)	MT272918 (identical to MT272915, MT272916,	MT272956 (identical to MT272953, MT272959, MT272957)
KS10/2646	Weissach	*Microtus arvalis*	2010 summer	MT272873 (identical to MT272869, MT272870, MT272871, MT272868, MT272875, MT272877, MT272886, MT272888, MT272872)	MT272919 (identical to MT272915, MT272916, MT272917, MT272918, MT272920)	MT272957 (identical to MT272953, MT272956, MT272959)

Table S6 (continued)

Isolate	Trapping location	Host	Collection date	S	M	L
KS10/3581	Weissach	*Microtus arvalis*	2010 fall	MT272874 (identical to MT272878, MT272881, MT272883, MT272884)	Identical to MT272922	MT272938
KS11/1955	Weissach	*Microtus arvalis*	2011 fall	MT272875 (identical to MT272869, MT272870, MT272871, MT272872, MT272868, MT272877, MT272886, MT272888)		MT272959 (identical to MT272953, MT272956, MT272957)
KS11/1998	Weissach	*Microtus arvalis*	2011 summer	MT272876		MT272960 (identical to MT272952)
KS11/2019	Weissach	*Microtus arvalis*	2011 summer	MT272877 (identical to MT272869, MT272870, MT272871, MT272872, MT272875, MT272868, MT272886, MT272888)	MT272920 (identical to MT272915, MT272916, MT272917, MT272918, MT272919)	MT272961
KS11/2289	Weissach	*Microtus arvalis*	2011 fall	MT272878 (identical to MT272874, MT272881, MT272883, MT272884)		MT272962 (identical to MT272966, MT272969)
KS11/2302	Weissach	*Microtus arvalis*	2011 fall	MT272879 (identical to MT272867)		MT272963
KS11/2305	Weissach	*Microtus arvalis*	2011 fall	MT272880 (identical to MT272886)	MT272921	MT272964
KS11/2313	Weissach	*Microtus arvalis*	2011 fall	MT272881 (identical to MT272878, MT272741, MT272883, MT272884)	MT272922	MT272965

Table S6 (continued)

Isolate	Trapping location	Host	Collection date	S	M	L
KS11/2314	Weissach	*Microtus arvalis*	2011 fall	MT272882 (identical to MT272885)	MT272923 (identical to MT272926, MT272924, MT272925)	MT272966 (identical to MT272969, MT272962)
KS11/2315	Weissach	*Microtus arvalis*	2011 fall	MT272883 (identical to MT272878, MT272881, MT272874, MT272884)	MT272924 (identical to MT272923, MT272926, MT272925)	MT272967
KS11/2318	Weissach	*Microtus arvalis*	2011 fall	MT272884 (identical to MT272878, MT272881, MT272883, MT272874)	MT272925 (identical to MT272923, MT272924, MT272926)	MT272968
KS11/2324	Weissach	*Microtus arvalis*	2011 fall	MT272885 (identical to MT272885)	MT272926 (identical to MT272923, MT272924, MT272925)	MT272969 (identical to MT272966, MT272962)
KS11/2325	Weissach	*Microtus arvalis*	2011 fall	MT272886 (identical to MT272869, MT272870, MT272871, MT272872, MT272875, MT272877, MT272878, MT272888, MT272880)		MT272970
KS12/2325	Weissach	*Microtus arvalis*	2012 summer	MT272887		
KS12/2338	Weissach	*Microtus arvalis*	2012 summer	MT272888 (identical to MT272869,		

Table S6 (continued)

Isolate	Trapping location	Host	Collection date	S	M	L
KS12/2338	Weissach	*Microtus arvalis*	2012 summer	MT272870, MT272871, MT272872, MT272875, MT272877, MT272886, MT272868)		
